# TokTogether: Harnessing TikTok’s Potential to Address Ageism in Health Education

**DOI:** 10.1093/geroni/igaf122.3863

**Published:** 2025-12-31

**Authors:** Kathryn Coccia

**Affiliations:** Saint Louis University, Indianapolis, Indiana, United States

## Abstract

**Background:**

Experts predict that the geriatric provider workforce needs to expand by over 100% by 2030 to meet projected population needs. Prior research has called for novel interventions to increase interest in geriatric specialization among health students.

**Methods:**

Surveying a sample of 110 interprofessional health students at Saint Louis University, this study tested the preliminary efficacy, feasibility, and acceptability of a novel intervention called TokTogether. Utilizing an explanatory mixed-methods design with an experimental group (n = 78) and a control group (n = 32), this study examined whether engaging with TikTok content created by and featuring older adults could effectively reduce participants’ ageism and age anxiety and increase interest in working with older adults (OAs)

**Results:**

Exposure to the TokTogether intervention significantly reduced ageism (t = 3.40, p = 0.001) and age anxiety (t = 4.80, p < 0.001) within the experimental group. It did not significantly increase interest in working with OAs (t = 0.79, p = 0.43). Participants in the experimental group showed a significantly greater reduction in ageism scores compared to those in the control group (t = 2.14, p = 0.03). Mixed-methods results supported the feasibility and acceptability of the intervention, with qualitative themes revealing that participants experienced increased feelings of connection with OAs, gained insight into the aging process, and reflected on and reevaluated their beliefs about aging and OAs.

**Conclusion:**

Engaging with TikTok content produced by and featuring older adults may be a scalable and effective way to reduce ageism and age anxiety among health students. Further investigation is needed about interventions to increase student interest in working with OAs.

